# The impact of the ovarian cycle on anxiety, allopregnanolone, and corticotropin releasing hormone changes after motherhood in female rats and women

**DOI:** 10.1038/s41398-023-02480-9

**Published:** 2023-05-30

**Authors:** Jodie E. Pestana, Kelly A. Kershaw, Bronwyn M. Graham

**Affiliations:** School of Psychology, UNSW Sydney, Sydney, NSW 2052 Australia

**Keywords:** Neuroscience, Human behaviour

## Abstract

Fluctuations in ovarian steroids across the estrous and menstrual cycle in female rats and women, respectively, are associated with changes in anxiety. Pregnancy causes long-term changes to ovarian hormone release, yet research on estrous- and menstrual-related changes in anxiety has focused on reproductively inexperienced females. Therefore, this study assessed whether the impact of estrous and menstrual cycles on anxiety differs pre- versus post-motherhood in female rats (*n* = 32) and a community sample of women (*n* = 63). Estrous cycle phase altered anxiety-like behavior in virgin rats, but had no effect in age-matched mother rats tested 1-month post-weaning. In humans, menstrual cycle phase was associated with ecological momentary assessed anxiety and mood in non-mothers, but not mothers; although, the menstrual cycle × reproductive status interaction for anxiety, but not mood, was rendered non-significant with age and cycle length as covariates. These findings suggest that changes in anxiety coincident with cycling hormones is an evolutionarily conserved feature of the estrous and menstrual cycle in rats and women, which is mitigated following motherhood in both species. We identified several potential mechanisms for the observed dissociation in estrous cycle effects on anxiety. Compared to virgin rats, mother rats had a lower peak and blunted decline in circulating allopregnanolone during proestrus, upregulated GABA_A_ receptor subunit (α1, α2, α5, α4, ß2) mRNA in the ventral hippocampus, and altered corticotropin-releasing hormone mRNA across the estrous cycle in the basolateral amygdala. Together, these findings suggest that the mechanisms underlying anxiety regulation undergo fundamental transformation following pregnancy in female rats and humans.

Anxiety and stressor-related disorders are twice as prevalent and more severe in women compared to men [1, 2]. Sex differences in the prevalence and burden of anxiety disorders typically emerge after puberty, suggesting that the menstrual cycle, and ensuing fluctuations in the ovarian steroids estradiol and progesterone, may contribute to the development and maintenance of anxiety disorders in women [3–6]. In addition, the menstrual cycle modulates the severity and expression of anxiety symptoms in a subset of hormonally sensitive healthy and anxious women, such that symptoms increase during menstrual phases of declining or low estradiol and progesterone levels (i.e., mid- to late-luteal phase and early-follicular phase, respectively) [7–9]. Conceptually consistent results occur across the rat estrous cycle, whereby anxiety-like behavior increases during estrous phases of low estradiol and progesterone (metestrus or diestrus) relative to those of high estradiol and progesterone (proestrus) [9, 10]. Administration of estradiol or progesterone and its metabolite allopregnanolone (a neurosteroid that acts as a positive allosteric modulator of the GABA_A_ receptor) reduces anxiety-like behavior in ovariectomized rats (chronically low estradiol and progesterone) and intact rats during diestrus [11–13]. However, abrupt (but not gradual) cessation of progesterone or allopregnanolone administration increases anxiety-like behavior, likely due to the effects of rapid withdrawal from allopregnanolone on the expression and function of GABA_A_Rs [14–16]. Together, these findings have led to the theory that *fluctuations* (i.e., increases and subsequent rapid decreases) in allopregnanolone across the ovarian cycle mediate cyclic-related changes in anxiety in female rodents and hormonally-sensitive humans [17, 18].

The impact of the ovarian cycle on anxiety has been examined in virgin female rodents and young women whose reproductive status is unknown; however, 85% of women will become a mother before the age of 44 [19]. Moreover, in rats and humans, reproductive experience (encompassing pregnancy, lactation, and maternal experience of caring for offspring) alters the nature and effects of ovarian steroid fluctuations across the ovarian cycle. For example, adult primiparous rats (one reproductive experience) have lower levels of circulating estradiol compared to nulliparous rats (no reproductive experience) during proestrus ([20, 21], but for opposite findings in middle-aged rats see [22]), and parous (reproductively experienced) women have lower estradiol levels than nulliparous women across the menstrual cycle [23, 24]. In addition, reproductive experience leads to long-term changes in brain regions related to anxiety, such as the amygdala and hippocampus [25]. For example, estrous cycle influenced GABA_A_R α2 subunit expression in the medial amygdala in nulliparous rats, but had no effect in primiparous rats [26]. GABA_A_R subunits are also modulated by estrous cycle in the hippocampus in nulliparous rats [27], however, no studies have assessed GABA_A_R subunit expression in the hippocampus in primiparous rats post-weaning, once cycling has recommenced. Reproductive experience leads to other long-term changes in the hippocampus, such as increased dendritic spine density and neurogenesis [25]. Such hormonal and neurobiological changes across the ovarian cycle following reproductive experience are accompanied by alterations in behavior on anxiety-related tasks. For instance, we have shown that reproductive experience mitigates effects of cycling ovarian steroids on fear extinction (a model of fear inhibition) in female rats and women [21].

Given that reproductive experience alters estradiol levels, anxiety-related gene expression, and behavioral fear inhibition across the ovarian cycle, we hypothesized that reproductive experience may alter the impact of the ovarian cycle on anxiety in females. To assess this hypothesis, we adopted a cross-species translational approach. In Experiment 1, we compared anxiety-like behavior during metestrus and proestrus in nulliparous and primiparous rats, and examined anxiety-related gene expression (including selective subunits of the GABA_A_R and corticotropin-releasing hormone, CRH) in the basolateral amygdala (BLA) and ventral hippocampus (vHPC), two brain regions critical for anxiety [28]. We found that reproductive experience mitigated the impact of estrous cycle on anxiety-like behavior in rats, which was likely due to pregnancy rather than the maternal experience during the postpartum period (Supplemental Experiment). Given that fluctuations in allopregnanolone have been implicated in cyclic-related changes in anxiety [18], and that allopregnanolone levels undergo dramatic changes across pregnancy, we hypothesized that reproductive experience might alter fluctuations in circulating allopregnanolone levels across the estrous cycle, as one potential mechanism that may mitigate estrous effects on anxiety-like behavior in primiparous rats. We confirmed this hypothesis in Experiment 2, in which we compared plasma allopregnanolone levels across various timepoints of the estrous cycle in nulliparous and primiparous rats. In Experiment 3, we demonstrated that the findings from female rats in Experiment 1 were translatable to nulliparous and parous women, such that menstrual cycle influenced self-reported anxiety and mood in non-mothers but not in mothers, suggesting the impact of pregnancy on the hormonal modulation of anxiety is conserved across species.

## Materials and methods

See the Supplemental Material for detailed methods.

### Animal subjects

Nulliparous and primiparous female Sprague Dawley rats obtained from the Animal Resources Centre (ARC), Australia were housed in groups of 5–8 at UNSW. Equal numbers of animals were allocated to each experimental group in each cage. The housing room was maintained on a 12-h light-dark cycle (lights on 0700), and food and water were available ad libitum. Mating to produce primiparous rats occurred at the ARC, as previously described [29]. Nulliparous and primiparous rats were age-matched at the time of each experiment, and underwent behavioral testing at approximately 6-months of age. Behavioral testing began in primiparous rats approximately 1-month after weaning, when lactation had ceased and estrous cycling had recommenced [21]. Sample sizes were determined according to previous investigations of estrous cycle effects on anxiety-like behavior [30–32]. All procedures were approved by the UNSW Animal Care and Ethics Committee.

### Estrous phase determination

Vaginal smears were conducted daily to determine estrous cycle phase, as previously described [33]. Only rats with a regular 4-day estrous cycle were included.

### Animal apparatus, histology, and procedures

In Experiment 1, rats were tested twice on a battery of anxiety tests including the light dark box (LDB), open field test (OFT), and elevated plus maze (EPM) over two testing days, once during metestrus and once during proestrus. The estrous phase on testing day was counterbalanced within reproductive groups. As the estrous phase is typically 4-days long, there was a 2-day rest period between testing days. For timeline, see Supplemental Fig. 1. The experimenter was blind to the experimental status of the animal during all scoring procedures.

#### Light-dark box

The LDB apparatus and procedure were administered as previously described [29]. Heightened anxiety-like behavior was indexed by a longer latency to enter the light compartment, a lower number of entries into the light compartment, and a shorter time spent in the light compartment [34]. The test lasted 5-min and occurred between 1200 and 1300 h.

#### Open field test

The OFT was a box (width = 60 cm, length = 60 cm, height = 50 cm) with a 36-square grid floor (6 × 6 squares, 10 cm/side), and an overhead light (144 lux) illuminating the central squares (all but the 20 perimeter squares were considered central). A video camera positioned above the apparatus was used to record animal behavior for later scoring. Heightened anxiety-like behavior was indexed by a shorter total time spent in the center of the open field and lower number of central crossings, whereas the number of peripheral crossings reflects differences in locomotor activity [35]. Rats were tested on the OFT one hour after the LDB between 1300 and 1400 h. The animal was placed in the central 4 squares and the test lasted 5-min.

#### Elevated plus maze

The EPM apparatus and procedure were administered as previously described [29]. Heightened anxiety-like behavior was indexed by a lower number of entries into the open arms and a shorter time spent in open arms, whereas the number of entries into the closed arms reflects locomotor activity [36]. An ‘anxiety index’ [37] was calculated using the following formula: 1 − [(time spent in open arms/test duration) + (open arm entries/total number of open and closed arm entries)/2]. A higher anxiety index score indicates higher anxiety-like behavior. Rats were tested on the EPM one hour after the OFT between 1300 and 1400 h, and the test lasted 5-min. Half the rats in each reproductive group underwent all three behavior tests, whereas the other half were tested on the EPM only (see Supplemental Information for details).

#### Tissue harvesting

One week after the EPM, rats were tested on fear conditioning and extinction paradigm (methods and data reported in [29]). Approximately one week later, rats were euthanized (0900–1100 h), during which acute changes in mRNA expression resulting from behavioral testing were no longer expected to be present. Equal numbers of the rats in each reproductive group were euthanized during metestrus and proestrus. Rats were euthanized following a brief exposure to Co2, and brains were rapidly removed and immediately frozen in liquid nitrogen [26]. Punches (2 mm in diameter, 1 mm in depth) from the BLA (ML = 4.9, AP = 2.5, DV = 8.4) and the vHPC (ML = 5, AP = 6, DV = 6) were taken based on coordinates determined by bregma [38].

#### Total RNA extraction and quantification

Total RNA was isolated from tissue punches using the Tri-Reagent® protocol (Sigma-Aldrich) according to manufacturer’s instructions. All samples met the required purity ratio criterion of >1.8 quantified using the NanoDrop Lite (Thermo Fisher Scientific). Messenger RNA was DNAse-treated (DNAse I Sigma-Aldrich) and first-strand cDNA was synthesized from 1 μg of total RNA using oligo(dt) random hexamer primers from the iScriptTM cDNA Synthesis Kit, in a T100 Thermal Cycler (Bio-Rad Laboratories).

#### Real-time quantitative polymerase chain reaction (RT-qPCR)

RT-qPCR gene expression analyses were performed in a StepOnePlus™ System (Applied Biosystems) using gene-specific TaqMan FAM/MGB assays (Applied Biosystems; Assays ID: GABAα1 ~ Rn00788315_m1, GABAα2 ~ Rn01413643_m1, GABAα4 ~ Rn00589846_m1, GABAα5 ~ Rn00568803_m1, GABAß2 ~ Rn00564149_m1, CRH~Rn01462137_m1, Rps18 ~ Rn01428913_gh, Sdha~Rn00590475_m1). Each PCR 10 μl reaction consisted of 4.5 μl of the sample cDNA (diluted 1:10 in nuclease-free H2O), 0.5 μl of the specific TaqMan assay, and 5 μl of TaqMan gene expression master mix (Applied Biosystems). Samples were run in triplicate and the expression level of target genes was normalized to the expression levels of two housekeeping genes (Rps18, Sdha).

#### Animal euthanasia and plasma allopregnanolone

In Experiment 2, we hypothesized that primiparous females may have a lower peak in circulating allopregnanolone during proestrus relative to nulliparous rats, as has been demonstrated for circulating estradiol [20, 21]. To capture both the rise and subsequent decline in allopregnanolone across proestrus, experimentally naïve nulliparous and primiparous rats were euthanized either during proestrus (9 am, 2 pm, or 6 pm) or estrus (9 am). These timepoints were chosen based on previous studies examining levels of hippocampal allopregnanolone [39] and peripheral progesterone [40] across the estrous cycle in nulliparous rats. Nulliparous and primiparous rats were also euthanized during metestrus (2 pm) to examine whether absolute *levels* of allopregnanolone differ between proestrus (2 pm) and metestrus (2 pm) at a similar timepoint to behavioral testing in Experiment 1 (when differences in anxiety-like behavior are typically apparent in nulliparous rats). Rats were euthanized following a brief exposure to Co2 and immediately decapitated with a guillotine. Trunk blood was rapidly collected in EDTA-treated tubes (Interpath Services, Victoria, Australia) and centrifuged within 30 min of collection.

Plasma was analyzed for allopregnanolone concentration using a commercially available ELISA kit, following manufacturer instructions (OKEH02604, Aviva Systems Biology, USA). Integrated optical density for each sample was measured using an iMARK plate reader (Bio-Rad Laboratories, Hercules, CA) at 450 nm. Data were extrapolated using four-parameter logistic regression software (myassays.com).

### Human participants

Naturally cycling nulliparous and parous women were recruited through local community advertisements. Eligibility criteria were as follows: aged 18–43 years, regular menstrual cycles (25–35 days), without medically diagnosed fertility problems, endocrine disorders, polycystic ovarian syndrome, or endometriosis, and had not used hormonal contraceptives, been pregnant or breastfeeding within the last 3 months. Sample and demographic information are reported in Table 1. Parous women had at least one biological child. Online informed consent was obtained from all the participants in accordance with the UNSW Human Research Ethics Committee (HC200234).

**Table 1:** Demographic and sample characteristics.

	Nulliparous women		Parous women		Statistic
	M (SD)	Range	M (SD)	Range	
Age (years)	23.77 (4.04)	18–39	32.64 (5.70)	20–43	*t*(61) = 7.22, *p* < 0.001
Menstrual cycle length (days)	30.54 (3.61)	21–42	28.71 (2.91)	23–36	*t*(107) = 2.86, *p* = 0.005
Time since last childbirth (years)	–	–	3.47 (2.80)	0.7–13	–
	***n***	**%**	***n***	**%**	
Race					χ2(3) = 21.14, *p* < 0.001
Caucasian	10	28.6	22	78.6	
Asian	23	65.7	4	14.3	
Aboriginal or Torres Strait Islander	0	0	2	7.1	
Other	2	5.7			
Marital status					χ2(2) = 34.55, *p* < 0.001
Never married	29	82.9	3	10.7	
Married/de-facto	6	17.1	23	82.1	
Separated/divorced/widowed	0	0	2	7.1	
Educational status					χ2(3) = 8.93, *p* = 0.04
Less than High School	0	0	1	3.6	
High School	16	45.7	4	14.3	
Technician, trade, or other certificate	3	8.6	9	32.1	
Tertiary (undergraduate)	10	28.6	10	35.7	
Tertiary (postgraduate)	6	17.1	4	14.2	
Employment status					χ2(2) = 16.96, *p* < 0.001
Full/part time paid work/self-employed	11	31.4	21	75	
Unemployed	3	8.6	4	14.3	
Student	21	69	3	10.7	
Number of pregnancies					
1	0	0	9	32.5	
2	0	0	14	50	
3	0	0	3	10.7	
4	0	0	1	3.6	
5	0	0	0	0	
6	0	0	1	3.6	
Number of biological children					
1	0	0	12	42.9	
2	0	0	15	53.6	
3	0	0	1	3.5	
Number of non-biological children					
1	0	0	25	89.3	
2	0	0	1	3.5	
3	0	0	2	7.2	
Self-reported postpartum anxiety/depression	0	0	18	64.3	

### Human materials and procedures

#### Patient health questionnaire 4 (PHQ-4)

The PHQ-4 is a 4-item scale used to measure anxiety and depression symptoms [41]. The wording in the PHQ-4 was adapted to present tense, such that participants were asked to rate the extent to which they were experiencing each item in the moment using a 4-point slider scale, *Not at all* (0–25), *Slightly* (25–50), *Moderately* (50–75), *Extremely* (75–100). It is common practice to adjust the wording of validated retrospective questionnaires to obtain a moment-to-moment assessment [42].

#### Procedure

Socio-demographic information was assessed via online surveys. General anxiety and mood symptoms were assessed using ecological momentary assessment (EMA). Participants were sent the PHQ-4 via text messages at three timepoints in the day: 9 am, 12 pm, and 9 pm, during three phases of the menstrual cycle spaced at 10-day intervals: phase 1 was the day after menses onset (estimated early follicular phase; low estradiol and progesterone), phase 2 was 12 days after menses onset (estimated mid-follicular phase; high estradiol and low progesterone), and phase 3 was 22 days after menses onset (estimated mid-luteal phase; high estradiol and progesterone). This procedure was repeated over two menstrual cycles (Supplemental Fig. 2). Due to individual variability in menstrual cycle length [43], the day count method (‘ovulation to ovulation’ backwards counting) was used post hoc to confirm the menstrual phase at the time of each testing phase for each participant [44, 45], which is the recommended method in the absence of measures of ovulation [46]; detailed description in Supplemental Information. The identified menstrual phases did not differ between nulliparous and parous women (Supplemental Table 1).

### Statistical analysis

The data were analyzed using SPSS, Version 22 (IBM, Armonk, NY, USA). In Experiment 1, two-way analyses of variances (ANOVAs) with repeated measures assessed group differences in anxiety-like behavior on the LDB, OFT, and EPM. To assess the hypothesis that estrous cycle would impact anxiety-like behavior in nulliparous but not primiparous rats, planned paired samples *t*-tests with Bonferroni correction (*p* = 0.025) examined estrous cycle effects on anxiety-like behavior in nulliparous and primiparous groups separately. Two-way ANOVAs were used to analyze group differences in mRNA expression within the BLA and vHPC. In Experiment 2, a two-way ANOVA assessed group differences in allopregnanolone concentration. In accordance our hypothesis, independent samples *t-*tests compared the rate of change in allopregnanolone concentration across proestrus (i.e., compared the rise and subsequent decline) in nulliparous and primiparous groups separately. In addition, to examine whether absolute *levels* of allopregnanolone differ between proestrus and metestrus at a similar timepoint to behavioral testing in Experiment 1, independent samples *t-*tests compared the allopregnanolone concentration between proestrus (2 pm) and metestrus (2 pm) in nulliparous and primiparous rats, separately. In Experiment 3, PHQ-4 scores were averaged for each item across timepoints (9am, 12 pm, 9 pm) in the day, and in each phase over the two menstrual cycles (e.g., estimated early follicular scores were calculated as an average of estimated early follicular scores during the first menstrual cycle and estimated early follicular scores during the second menstrual cycles). The PHQ-4 was split into an anxiety (average of item 1 and 2) and depression (average of item 3 and 4) subscale [41]. Two-way ANOVAs with repeated measures assessed scores over the menstrual cycle on the dependent variables PHQ-anxiety and PHQ-depression. Significant interactions were followed up using one-way ANOVAs, and significant main effects using paired-sample *t*-tests. The Greenhouse–Geisser correction was used if the assumption of sphericity was violated (as indicated by Mauchly’s Test of Sphericity). In Experiment 1, seven out of 198 data points across all target genes in the BLA and 20 out of 234 data points across all target genes in the vHPC were excluded due to being statistical outliers (defined as 2 s.d. away from mean). One statistical outlier was removed from Experiment 2 (details in Supplemental Information).

## Results

### Experiment 1: Reproductive experience attenuates the impact of estrous cycle on anxiety-like behavior in female rats

#### LDB

**Fig. 1:**
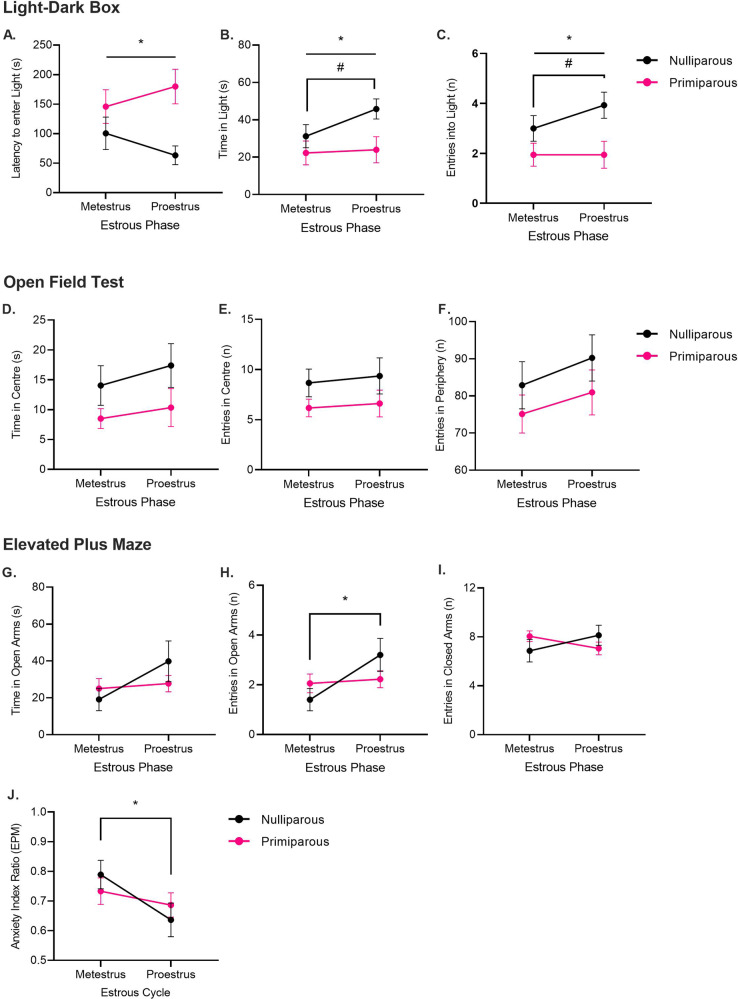
Reproductive experience attenuates estrous cycle effects on anxiety-like behavior in female rats. In Experiment 1, nulliparous and primiparous rats were tested twice on the light-dark box (LDB), open field test (OFT), and elevated plus maze (EPM); once during metestrus, and once during proestrus, with the estrous phase at test being counterbalanced. **A** Mean (±SEM) latency to enter the light compartment in the LDB in nulliparous rats (*n* = 14) and primiparous rats (*n* = 18). *Primiparous rats > Nulliparous rats (*p* < 0.05). **B** Mean (±SEM) time spent in the light compartment. *Primiparous rats < Nulliparous rats (*p* < 0.025); #Nulliparous-Metestrus < Nulliparous-Proestrus (*p* < 0.05). **C** Mean (±SEM) number of entries into the light compartment. *Primiparous rats < Nulliparous rats (*p* < 0.025). #NulliparousMetestrus < Nulliparous-Proestrus (*p* < 0.05). **D** Mean (±SEM) time spent in the center squares of the OFT in nulliparous rats (*n* = 14) and primiparous rats (*n* = 18). **E** Mean (±SEM) number of entries in the center squares. **F** Mean (±SEM) number of entries in the periphery squares. **G** Mean (±SEM) time spent in open arms of the EPM in nulliparous rats (*n* = 19) and primiparous rats (*n* = 18). **H** Mean (±SEM) number of entries in open arms. *Nulliparous-Metestrus < Nulliparous-Proestrus (*p* < 0.025). **I** Mean (±SEM) number of entries in the closed arms. **J** Mean (±SEM) anxiety index ratio. *NulliparousMetestrus < Nulliparous-Proestrus (*p* < 0.025).

There were no main effects of estrous phase or estrous phase × reproductive status interactions on any measure (Fig. 1A–C, largest *F*_(1,30)_ = 3.13, *p* = 0.09). However, there was a main effect of reproductive status on all measures whereby primiparous females spent less time in the light compartment (*F*_(1,30)_ = 4.21, *p* = 0.049), made fewer entries in the light compartment (*F*_(1,30)_ = 6.85, *p* = 0.01), and had a longer latency to enter the light compartment (*F*_(1,30)_ = 6.57, *p* = 0.02), compared to nulliparous females. Nulliparous females tended to show an increase in the time spent in the light compartment (*t*_(13)_ = 2.29, *p* = 0.04, *d* = 0.61) and number of entries in the light compartment (*t*_(13)_ = 2.18, *p* = 0.048, *d* = 0.58) from metestrus to proestrus, however, these effects did not withstand Bonferroni corrections. The latency to enter the light compartment remained unchanged (*t*_(13)_ = 1.71, *p* = 0.11, *d* = 0.46). In primiparous rats, all behaviors remained unchanged from metestrus to proestrus (largest *t*_(17)_ = 1.10, *p* = 0.29, *d* = 0.26).

#### OFT

**Fig. 2:**
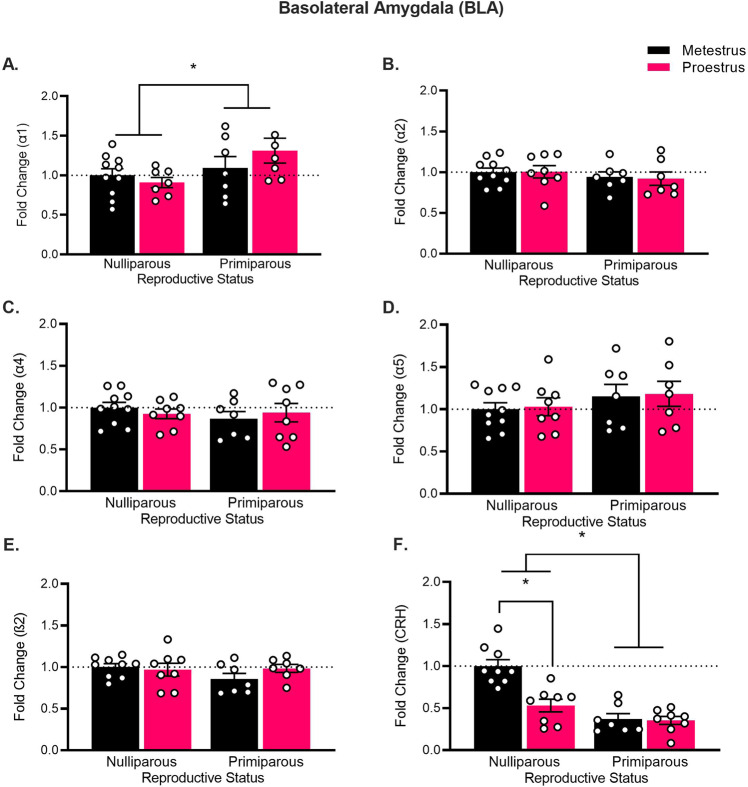
Reproductive experience alters anxiety-related gene expression in the basolateral amygdala. Mean (±SEM) gene expression of **A** GABAAR α1 subunit, **B** GABAAR α2 subunit, **C** GABAAR α4 subunit, **D** GABAAR α5 subunit, **E** GABAAR ß2 subunit, and **F** CRH, within the basolateral amygdala in nulliparous and primiparous rats, euthanized during either metestrus or proestrus (*n* = 7–10). Primiparous rats had higher α2 subunit expression than nulliparous rats. Primiparous rats had lower CRH expression than nulliparous rats. Nulliparous-metestrus rats had higher CRH expression than nulliparous-proestrus rats (*ps < 0.05).

There were no main effects of estrous phase or reproductive status, and no estrous phase × reproductive status interactions on any measure (Fig. 2D–F; largest *F*_(1,30)_ = 3.03, *p* = 0.09). The number of peripheral and central entries, as well as time spent in the central squares remained unchanged from metestrus to proestrus in both nulliparous and primiparous rats (*t*s < 1).

#### EPM

In the number of open arm entries, there was a main effect of estrous phase (Fig. 1G–J; *F*_(1,35)_ = 8.57, *p* = 0.006) and an estrous phase × reproductive status interaction (*F*_(1,35)_ = 5.25, *p* = 0.03) but no main effect of reproductive status (*F* > 1). There were no main effects or interaction between factors in the time spent in the open arms (largest *F*_(1,35)_ = 3.52, *p* = 0.07). In the number of closed arm entries, there were no main effects (*F*s > 1) but there was an estrous phase × reproductive status interaction (*F*_(1,35)_ = 6.41, *p* = 0.02). Nulliparous females showed an increase in the number of open arm entries from metestrus to proestrus (*t*_(18)_ = 3.11, *p* = 0.01, *d* = 0.71), and a similar increase in the time sent in the open arms but this did not reach significance (*t*_(18)_ = 1.92, *p* = 0.07, *d* = 0.44). The number of closed entries remained unchanged (*t*_(18)_ = 1.65, *p* = 0.12, *d* = 0.38). In primiparous females, the number of closed arm entries tended to decrease from metestrus to proestrus, but this did not reach significance (*t*_(17)_ = 2.01, *p* = 0.06, *d* = 0.47) and all open arm measures remained unchanged (*t*s < 1, *d*s < 0.15).

In the anxiety index, there was a main effect of estrous phase (*F*_(1,35)_ = 9.66, *p* = 0.004), but no main effect of reproductive status (*F* < 1) or interaction between factors (*F*_(1,35)_ = 2.74, *p* = 0.12). Nulliparous females showed a decrease in the anxiety index from metestrus to proestrus (*t*_(18)_ = 3.04, *p* = 0.01, *d* = 0.70). In contrast, primiparous rats showed no change in the anxiety index from metestrus to proestrus (*t*_(17)_ = 1.19, *p* = 0.25, *d* = 0.28). Inclusion of battery of tests as a covariate in each analysis led to the main effect of estrous phase to be non-significant in the open arm entries and anxiety index ratio (*F*s < 1), however, the estrous phase × reproductive status interactions remained significant in the open arm and closed arm entries (Supplemental Results). These findings in primiparous rats were replicated in a follow-up experiment, in which we also demonstrated that anxiety-like behavior on the EPM was estrous-cycle-independent even in primiparous rats that had their pups removed within 24 h following birth (Supplemental Experiment).

#### BLA gene expression

Primiparous rats had higher overall α1 mRNA expression compared to nulliparous rats (Fig. 2; main effect of reproductive status, *F*_(1,28)_ = 5.82, *p* = 0.02) but there was no main effect of estrous phase and no estrous phase × reproductive status interaction (largest *F*_(1,28)_ = 2.92, *p* = 0.10). There were no group differences in α2, α4, α5, and ß2 subunits (no main effects or interaction; largest *F*_(1,27)_ = 2.68, *p* = 0.11). Primiparous rats had lower overall CRH mRNA (main effect of reproductive status, *F*_(1,28)_ = 34.90, *p* < 0.001). There was also a main effect of estrous phase (*F*_(1,28)_ = 12.76, *p* = 0.001) and a reproductive × estrous phase interaction in CRH mRNA (*F*_(1,28)_ = 11.01, *p* < 0.01). Follow-up *t-*tests revealed that nulliparous-metestrus rats had higher CRH mRNA than nulliparous-proestrus rats (*t*_(15)_ = 4.38, *p* < 0.001, *d* = 2.13), whereas primiparous-metestrus and primiparous-proestrus rats did not differ (*t*_(13)_ = 0.22, *p* = 0.83, *d* = 0.11).

#### vHPC gene expression

**Fig. 3:**
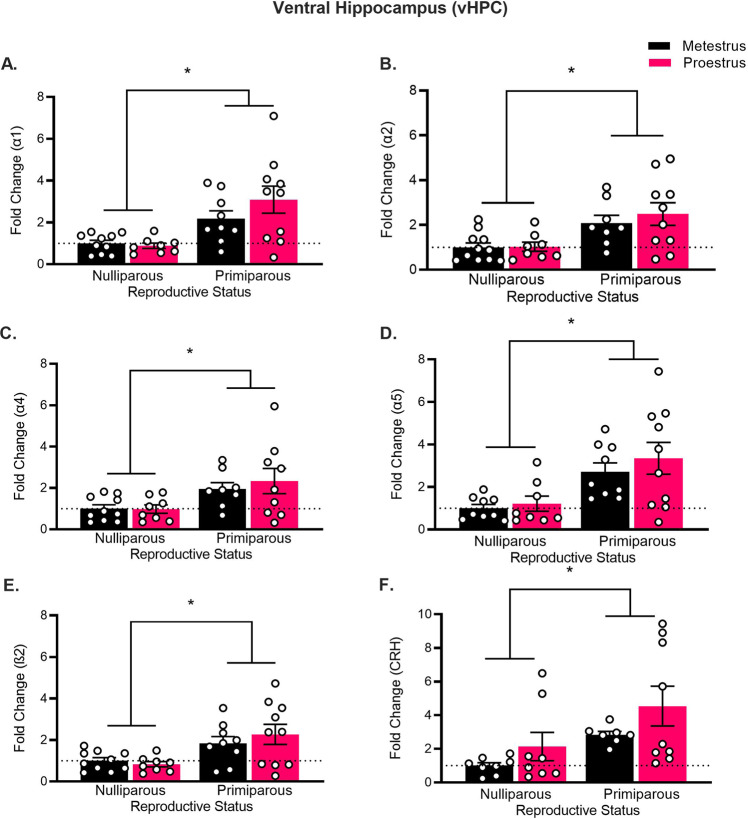
Reproductive experience alters anxiety-related gene expression in the ventral hippocampus. Mean (±SEM) gene expression of **A** GABAAR α1 subunit, **B** GABAAR α2 subunit, **C** GABAAR α4 subunit, **D** GABAAR α5 subunit, **E** GABAAR ß2 subunit, and **F** CRH, within the ventral hippocampus in nulliparous and primiparous rats, euthanized during either metestrus or proestrus (*n* = 8–10). Primiparous rats had higher mRNA expression of GABAAR subunits α1, α2, α4, α5, and ß2, and CRH than nulliparous rats (*ps < 0.05).

There was a main effect of reproductive status in α1 (Fig. 3; *F*_(1,33)_ = 16.96, *p* < 0.001), α2 (*F*_(1,33)_ = 13.26, *p* < 0.001), α4 (*F*_(1,31)_ = 9.52, *p* < 0.01), α5 (*F*_(1,32)_ = 14.63, *p* < 0.001) and ß2 (*F*_(1,33)_ = 12.49, *p* < 0.001) subunits, whereby primiparous rats exhibited higher mRNA expression of all GABA_A_ subunits compared to nulliparous rats, but there were no main effects of estrous phase or estrous phase × reproductive interactions (largest *F*_(1,33)_ = 1.56, *p* = 0.22). Primiparous females also had higher CRH mRNA than nulliparous females (main effect of reproductive status; *F*_(1,28)_ = 6.97, *p* = 0.01), but there was no main effect of estrous phase or estrous phase × reproductive status interaction (largest *F*_(1,28)_ = 3.13, *p* = 0.09).

### Experiment 2: Reproductive experience attenuates the peak and blunts the subsequent decline of plasma allopregnanolone during proestrus in female rats

**Fig. 4:**
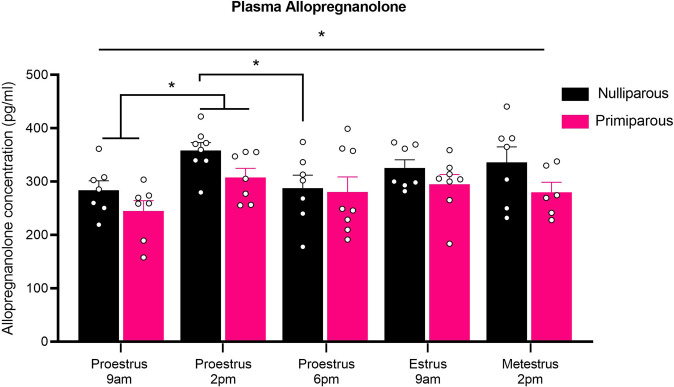
Reproductive experience alters allopregnanolone levels during proestrus in female rats. Mean (±SEM) plasma allopregnanolone concentration in nulliparous rats at proestrus 9 am (*n* = 8), proestrus 2 pm (*n* = 8), proestrus 6 pm (*n* = 7), estrus 9 am (*n* = 7), and metestrus 2 pm (*n* = 7), as well as primiparous rats at proestrus 9 am (*n* = 7), proestrus 2 pm (*n* = 7), proestrus 6 pm (*n* = 8), estrus 9 am (*n* = 8), and metestrus 2 pm (*n* = 6). Nulliparous rats had higher allopregnanolone than primiparous rats. Nulliparous rats had higher allopregnanolone at proestrus 2 pm compared to 9 am and 6 pm. Primiparous rats had higher allopregnanolone at 2 pm compared to 9 am (*p’s < 0.05).

There was a main effect of reproductive status (Fig. 4; *F*_(1,62)_ = 7.62, *p* = 0.01), such that allopregnanolone concentration across all timepoints was reduced by 11.75% in primiparous rats, with a mean of 319.51 pg/mg in nulliparous rats and a mean of 281.96 pg/ml in primiparous rats. This effect was primarily driven by group differences at proestrus 2 pm, as nulliparous rats had significantly higher allopregnanolone concentration compared to primiparous rats at proestrus 2 pm (*t*_(13)_ = 2.27, *p* = 0.04), but groups did not differ at any other timepoint (largest *t*_(12)_ = 1.57, *p* = 0.14). There was also a main effect of estrous phase timepoint (*F*_(4,62)_ = 3.17, *p* = 0.02) but no reproductive status × estrous phase timepoint interaction (*F* < 1). Regarding the rate of change in allopregnanolone across proestrus, nulliparous rats showed a significant 26.19% increase in allopregnanolone concentration from 9 am to 2 pm (*t*_(13)_ = 3.29, *p* = 0.01), and then a significant 25.69% decrease from 2 pm to 6 pm (*t*_(13)_ = 2.55, *p* = 0.02). In contrast, primiparous rats showed a significant 19.79% increase in allopregnanolone concentration from 9 am to 2 pm (*t*_(12)_ = 2.42, *p* = 0.03), however, they showed no significant change from 2 pm to 6 pm (non-significant decrease by 8.83%; *t*_(13)_ = 0.82, *p* = 0.43). There was no difference between proestrus (2 pm) and metestrus (2 pm) in either nulliparous (*t*_(13)_ = 0.69, *p* = 0.51) or primiparous (*t*_(11)_ = 1.01, *p* = 0.30) groups.

### Experiment 3: Reproductive experience attenuates the impact of menstrual cycle on anxiety and mood symptoms in humans

#### PHQ-4 anxiety

**Fig. 5:**
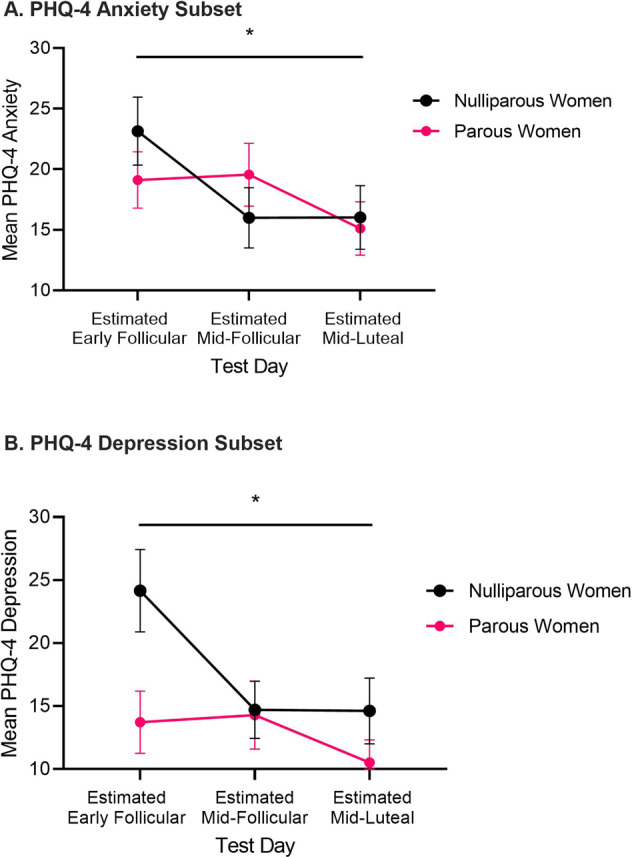
Reproductive experience attentuates menstrual cycle effects on anxiety and mood symptoms in humans. Mean (±SEM) scores across phases of the menstrual cycle in nulliparous (*n* = 35) and parous women (*n* = 28) on the **A** anxiety subset of the PHQ-4, and **B** depression subset of the PHQ-4. Estimated early follicular phase (phase 1) is the day after menses onset (low estradiol and low progesterone levels), estimated mid-follicular phase (phase 2) is 12 days after menses onset (estimated high estradiol and low progesterone levels), and estimated mid-luteal phase (phase 3) is 22 days after menses onset (estimated high estradiol and high progesterone levels). Nulliparous women self-reported higher anxiety and depression at the estimated early follicular phase compared to the estimated mid-follicular and mid-luteal phases (*p*’s < 0.05).

There was a main effect of menstrual cycle (Fig. 5A; *F*_(2,122)_ = 6.70, *p* < 0.01), and a menstrual cycle × reproductive status interaction (*F*_(2,122)_ = 3.10, *p* = 0.049), and no main effect of reproductive status (*F* < 1). In nulliparous women, there was a main effect of menstrual cycle (*F*_(2,68)_ = 8.93, *p* < 0.001). Anxiety scores were higher at the estimated early follicular phase compared to the estimated mid-follicular phase (*t*_(34)_ = 3.34, *p* = 0.01) and estimated mid-luteal phase (*t*_(34)_ = 3.34, *p* < 0.01). There was no difference in anxiety scores between the estimated mid-follicular and mid-luteal phases (*t* < 1). In parous women, there was no main effect of menstrual cycle (*F*_(2,54)_ = 2.07, *p* = 0.14).

#### PHQ-4 depression

There was a main effect of menstrual cycle (Fig. 5B; *F*_(2,122)_ = 7.66, *p* = 0.001), and a menstrual cycle × reproductive status interaction (*F*_(2,122)_ = 4.61, *p* = 0.02), and no main effect of reproductive status (*F*_(1,61)_ = 2.45, *p* = 0.12). In nulliparous women, there was a main effect of menstrual cycle (*F*_(2,68)_ = 11.40, *p* < 0.001). Depression scores were higher at the estimated early follicular phase compared to the estimated mid-follicular phase (*t*_(34)_ = 3.35, *p* = 0.002) and estimated mid-luteal phase (*t*_(34)_ = 4.32, *p* < 0.001). There was no difference in depression scores between the estimated mid-follicular and mid-luteal phases (*t* < 1). In parous women, there was no main effect of menstrual cycle (*F*_(2,54)_ = 1.45, *p* = 0.24).

#### Covariate analyses

Inclusion of age and menstrual cycle length as covariates removed the menstrual cycle × reproductive status interaction for anxiety, but not for depression. The time since childbirth, or multiparity (i.e., having had 2 or more biological children) did not moderate menstrual cycle effects on anxiety or depression (Supplemental Results).

## Discussion

These experiments demonstrate that reproductive experience may mitigate cyclic-related changes in anxiety in female rats and women; effects that persist long after the hormonal surges of pregnancy and lactation have diminished. Replicating previous findings [9, 10], nulliparous rats showed heightened anxiety-like behavior during metestrus compared to proestrus on the EPM, and a trend in the same direction on the LDB (effect sizes ranging from medium to large). In contrast, no estrous effects were observed on the OFT. This null finding is consistent with previous mixed findings on this task [30–32, 47, 48]. Irrespective, the novel finding is that primiparous rats showed comparable anxiety-like behavior during metestrus and proestrus on all three tasks, with effect sizes ranging from negligible to small. These findings in rats translated to humans—in contrast to nulliparous women who prospectively reported worse anxiety and mood during the early follicular phase compared to the mid-follicular and mid-luteal phases, parous women reported comparable anxiety and mood symptoms across menstrual cycle phases. However, the menstrual cycle × reproductive status interaction in anxiety was rendered non-significant after including age and cycle length as covariates, which raises the possibility that cyclic-related changes in anxiety in nulliparous versus parous women may be due to differences in age or cycle length rather than their reproductive history. However, nulliparous and primiparous rats were aged-matched and experienced a regular 4–5 day estrous cycle suggesting that mitigation of cyclic-related changes in anxiety may not be completely attributed to age and cycle length differences, at least in female rats. The exacerbation of symptoms in nulliparous women in the early follicular phase is consistent with past studies [49–53], which likely reflect a continuation of symptoms that begin to rise in the mid to late luteal phase [51, 54–56]. In our study, the vast majority of participants were assessed earlier in the luteal phase, from ovulation to mid-luteal phase, when ovarian steroids are expected to still be high, paralleling the proestrus phase examined in rats. Thus, it will be important for future research to compare symptoms experienced during the late-luteal phase in parous and nulliparous women.

Combined, these findings demonstrate that cyclic-related changes in anxiety may be an evolutionarily conserved feature of the ovarian cycle in rats and humans, but that this effect may be mitigated post-reproductive experience in both species. Such effects are likely due to the hormonal surges associated with pregnancy, rather than maternal experience during the postpartum period, as primiparous rats that experienced pregnancy but had their pups removed within 24 h after birth also showed estrous-cycle-*independent* anxiety-like behavior (Supplemental Experiment). Moreover, in humans, these effects did not depend on the number of pregnancies, or the time since childbirth, raising the possibility the effects in primiparous rats may also persist beyond 1-month post-weaning assessed in primiparous rats.

The conceptually consistent findings in female rats and humans suggest that similar cross-species mechanisms may mediate cyclic-related changes in anxiety, which are then altered by pregnancy in both species. We identified several candidate mechanisms in rats. Reproductive experience led to a lower peak in allopregnanolone during the afternoon of proestrus, and a subsequent blunted and non-significant decline during the evening of proestrus. In nulliparous rats, the rapid decline in allopregnanolone during the evening of proestrus may result in the withdrawal-like increase in anxiety-like behavior during metestrus. In support of this hypothesis, the abrupt cessation of an exogenous progesterone dosing regimen leads to increases in anxiety-like behavior [16, 57–61], effects which depend on progesterone’s conversion to allopregnanolone [16]. In contrast to the abrupt cessation of progesterone, a gradual decline in exogenous progesterone has no effect on anxiety-like behavior [59, 60]. Therefore, one account for why primiparous females exhibit estrous-cycle-*independent* anxiety-like behavior is that the *gradual decline* in allopregnanolone during the evening of proestrus prevents withdrawal-like effects during metestrus. Future studies could examine the impact of increasing allopregnanolone in primiparous rats during proestrus on anxiety-like behavior 48 h later during metestrus. No studies have assessed whether reproductive experience alters allopregnanolone levels across the menstrual cycle in humans. However, reproductive experience causes cross-species reductions in estradiol across the ovarian cycle [20, 21, 23, 24]. As estradiol upregulates allopregnanolone synthesis [62, 63], reduced levels of estradiol across the ovarian cycle in rats and humans may lead to lower allopregnanolone synthesis in both species. Given estradiol’s involvement in anxiety regulation [11], it is also possible that reductions in estradiol across the ovarian cycle may mitigate cyclic-related changes in anxiety following reproductive experience across species e.g., via downstream effects on estrogen nuclear receptors [64].

Another mechanism that may contribute to the mitigation of cyclic-related changes in anxiety following reproductive experience is alterations in the neural circuitry of anxiety. The behavioral findings in female rats in the current study aligned with changes in CRH mRNA expression in the BLA, whereby nulliparous rats showed higher CRH expression during metestrus compared to proestrus, whereas primiparous rats showed comparable CRH expression irrespective of estrous phase. CRH, particularly within the extended amygdala circuitry, produces anxiogenic-like states [65–68]. As such, it is possible that higher CRH expression in the BLA during metestrus versus proestrus in nulliparous rats may, in part, lead to their heightened anxiety-like behavior during metestrus, potentially due to increased CRH projections within the extended amygdala such as central amygdala and bed nucleus of the stria terminalis [65]. In contrast, as CRH expression in the BLA did not differ between metestrus and proestrus in primiparous females, this may account for their comparable anxiety-like behavior across estrous phases. Compared to nulliparous rats, primiparous rats also had lower overall CRH expression in the BLA but higher overall CRH expression in the vHPC, suggesting that reproductive experience may produce site-specific changes in CRH expression. Moreover, reproductive experience led to an upregulation of all GABA_A_R subunits examined (α1, α2, α5, α4, ß2) in the vHPC. These parity-induced changes in the vHPC were not due to global effects on GABA_A_R subunits throughout the brain given that only α1 expression was upregulated in the BLA. Changes in the GABA_A_R subunit composition in the hippocampus may influence anxiety by affecting ion gradients, and in turn, excitability of the neurons expressing GABA_A_Rs [69].

Limitations of the present experiments include self-report assessment of anxiety and mood in humans, which is subject to reporting bias; although EMA is considered the gold-standard method with which to reduce retrospective reporting biases [42]. In addition, due to COVID-19 restrictions, we could not measure ovarian steroids or confirm ovulation in humans. Moreover, we did not assess for current psychiatric diagnoses or medication use in our human community sample. Although there is no evidence to suggest that ovarian steroid levels differ between those with versus those without psychiatric diagnosis, it is possible that menstrual cycle effects on mood and anxiety in one group could have been obscured by ceiling effects if there was a disproportionate number of individuals with severe psychiatric symptoms in that group. Finally, mRNA levels were measured in female rats that had prior exposure to anxiety-eliciting tasks so whether similar patterns in CRH and GABA_A_R subunit expression are evident at baseline in experimentally naïve animals is unknown.

The present findings, coupled with our previous demonstrations of profound effects of reproductive experience on fear extinction [21], raise the possibility that the impact of the menstrual cycle on anxiety disorders may shift following reproductive experience. While the menstrual cycle impacts the expression and maintenance of anxiety disorders in nulliparous women [7], our findings suggest this may not be the case in women following reproductive experience. In addition, these findings raise the possibility that the efficacy of anxiolytic drugs may also differ as a function of reproductive status. Selective serotonin reuptake inhibitors (SSRIs) increase brain allopregnanolone levels which leads to anxiolytic effects in rats, and benzodiazepines target GABA_A_Rs [70, 71], both of which were altered in primiparous rats. Moreover, compared to nulliparous rats, primiparous rats post-weaning showed greater sensitivity to the anxiolytic effects of the benzodiazepine, diazepam [72]. In addition, compared to nulliparous rats, primiparous rats during the postpartum period are resistant to some effects of fluoxetine (an SSRI) and 8-OH-DPAT (a serotonergic agonist) [73, 74]. Finally, recent reviews have outlined individual difference factors that may contribute to mixed results on menstrual cycle effects on anxiety in humans (e.g., individual differences in hormonal sensitivity or health anxiety; [7–9]). The current results suggest that reproductive status may be another individual difference factor contributing to mixed results across studies that could be accounted for in future studies.

## Supplementary information

Supplemental Information
