# Supplementary material for: The impact of the ovarian cycle on anxiety, allopregnanolone, and corticotropin releasing hormone changes after motherhood in female rats and women
Source: Transl Psychiatry. 2023 May 30;13:183. doi: 10.1038/s41398-023-02480-9 (PMC10228440; doi:10.1038/s41398-023-02480-9)
Supplement: Supplementary file 1 — Supplemental Information [file 41398_2023_2480_MOESM1_ESM.docx]

**The impact of the ovarian cycle on anxiety, allopregnanolone, and corticotropin releasing hormone changes after motherhood in female rats and women**

***Supplemental Information***

**Supplemental Methods and Materials - Experiments Reported in Manuscript**

***Animal Subjects***

At UNSW, rats were housed in groups of 5-8 in plastic boxes (67 cm long x 30 cm wide x 22 cm high) filled with corncob bedding and covered with a wire lid. The boxes were kept inside a colony room that was maintained at 20–22°C on a 12-hour light-dark cycle (lights on at 0700). Food and water were available ad libitum and rats remained under these conditions for an acclimatization period of 2 weeks prior to the commencement of any procedures. All experimental procedures occurred during the light phase, in line with previous studies examining anxiety-like behaviour in naturally cycling female rats (1, 2, 3, 4, 5). All rats were treated according to The Australian Code of Practice for the Care and Use of Animals for Scientific Purposes (8th edition, 2013), and all procedures were approved by the Animal Care and Ethics Committee at UNSW.

***Animal Vaginal cytology***

Starting on the first day of handling, vaginal cytology was assessed daily between 0900h and 1100h to ensure regular estrous cycling across experiments; a regular 4-5 day estrous cycle consists of four separate phases: metestrus, diestrus, proestrus, and estrus. Rats were placed in an open plastic box (24.5 cm long x 37 cm wide x 27 cm high) containing saw dust bedding, and a cotton-tip moistened with 0.9% saline was inserted into the vaginal canal. The cotton tip was twisted to collect loose epithelial cells. The cells were transferred to a microscope slide and dyed with KwikDiff Stain Kit before being observed under a light microscope (x10). Estrous phase was determined by the presence of different types of cells (6): nucleated cells (proestrus), cornified cells (estrus), and leukocytes (diestrus), as well as the absence of leukocytes (metestrus).

***Experiment 1 Procedure***

**Handling**. Rats were handled for 5 min and weighed and swabbed each day for three consecutive days prior to any experimental procedures.

**Battery of tests.** Rats were tested twice on three unlearned fear tasks including the LDB, OFT, and EPM, over two testing days, once during metestrus and once during proestrus. On testing days, rats were individually exposed to each unlearned fear task in a separate testing room, while the remaining rats stayed in their home cage in the laboratory corridor. Note that the order of testing was the same for each rat so that if prior testing on a task confounded performance on a subsequent task, then the impact would be the same for each rat. This procedure was based on previous studies that tested rats on multiple unlearned fear tasks on the same day (7, 8, 9, 10, 11, 12). Following each task, rats were immediately returned to their home cage for a 1-hour rest period. The purpose of this rest period was to allow stress elicited by each task to subside before the same rat was tested on the subsequent task. Although placing the animal back in their home cage could introduce variance in anxiety-like behaviour in cage mates due to emotional contagion, this procedure was necessary as it avoided the effects of social isolation on subsequent anxiety-like behaviour. Rats were transported to and from testing rooms in a yellow bucket containing corncob bedding. The testing procedure of each unlearned fear task is described below.

***Light-dark box.*** Rats were tested on the LDB between 1200h-1300h. At test, the animal was confined in the dark compartment for 1-min by blocking the opening with a sliding metal door. The experimenter then removed the door which allowed access to the light compartment for 5-min. The door of the testing room was closed to minimise external noise and visual disruption, and the experimenter observed the animals’ behaviour in an adjacent room. Video recordings of the animals’ behaviour were scored by the experimenter after behavioural testing was complete. During scoring, the experimenter was blind to the experimental status of each animal. The LDB was cleaned with 70% ethanol between tests.

***Open field test*.** One hour after the LDB, rats were tested on the OFT between 1300h-1400h. The animal was placed in the central 4 squares and observed for 5 min. The door of the testing room was closed to minimise external noise and visual disruption, and the experimenter observed the animals’ behaviour in an adjacent room. Video recordings of the animals’ behaviour were scored by the experimenter after behavioural testing was complete. During scoring, the experimenter was blind to the experimental status of each animal. The OFT was cleaned with 70% ethanol between tests.

***Elevated plus maze.*** One hour after the OFT, rats were tested on the EPM between 1400h-1500h. The animal was placed in the centre of the maze facing an open arm and observed for 5 min. The door of the testing room was closed to minimise external noise and visual disruption, and the experimenter observed the animals’ behaviour in an adjacent room. Video recordings of the animals’ behaviour were scored by the experimenter after behavioural testing was complete. During scoring, the experimenter was blind to the experimental status of each animal. The EPM was cleaned with 70% ethanol between tests.

Because illumination influences performance on the EPM in female rats (5), each rat was initially tested twice on the EPM on the same testing day under two illumination conditions: low light intensity (5 lux) and high light intensity (300 lux). The illumination level was created by the absence or presence of a ceiling light. However, after testing 18 rats (n = 8 nulliparous rats, n = 10 primiparous rats) on both lighting conditions, it became apparent that the low light intensity resulted in ceiling effects, such that rats were spending an equal amount of time in the open arms and closed arms, and therefore could not distinguish between these two conditions. In contrast, the high light intensity resulted in no such ceiling effects, indicated by the smaller amount of time spent in the open compared to the closed arms. Therefore, all the rats that were tested under both low and high light intensity were excluded from the analysis of the EPM. Note that these rats remained in the analysis of the LDB and OFT as these tests occurred prior to the EPM. To make up the numbers for the EPM, additional animals were tested on the EPM under the high light intensity only. As such, half the nulliparous and primiparous rats included in Experiment 1 underwent all three behaviour tests, whereas half the rats were tested on the EPM only. The results in the EPM remained similar when battery of tests was included as a covariate (see Supplemental Results). All rats included in the analysis underwent fear conditioning and extinction 7 days later (data reported in 13).

**Tissue harvesting.** Rats were euthanised approximately one week after conditioning/extinction procedures, during which acute changes in mRNA expression resulting from behavioural testing were no longer expected to be present. Following euthanasia and brain extraction, whole brains were stored at -80°C until brain dissection. Brain punches were taken in a cryostat maintained at -20°C. Punch samples were added to 1.5ml microtubes containing 25 μg of glass beads, and stored at -80°C prior to total RNA extraction.

**Total RNA quantification**. NanoDrop Lite (Thermo Fisher Scientific) spectrophotometry at 260 nm was used to quantify the total RNA concentration (ng/μl) in each sample and their purities were measured by determining the absorption ratios at 260/280 nm. All samples met the required purity ratio criterion of >1.8.

**Gene Expression.** We measured anxiety-related gene expression including selective subunits of the GABA_A_ receptor, as the major inhibitory component of the central nervous system. Reproductive experience alters selective subunits of the α subunit family (14). For example, mRNA expression of the α2 subunit was higher during proestrus compared to diestrus within the medial amygdala in nulliparous rats, but did not differ between estrous phases in primiparous rats at 4 weeks post-weaning. Primiparous rats also had higher overall α2 subunit mRNA expression within the periaqueductal gray compared to nulliparous rats (14). We aimed to extend these previous findings by examining whether reproductive experience alters α subunits within the BLA and vHPC, two key brain regions involved in anxiety regulation. We assessed expression of benzodiazepine-sensitive subunits (α1, α2, α5) and a benzodiazepine-insensitive subunit (α4) from the α subunit family, as well as the ß2 subunit from the ß subunit family, to assess whether potential changes in GABA_A_ receptor subunit expression following reproductive experience were specific to the subunit type and/or α subunit family, respectively. In addition, we assessed corticotropin releasing hormone (CRH) as CRH is the primary activator of the HPA axis stress response and has been implicated in anxiety in rats and humans (15, 16). Moreover, the CRH system undergoes dramatic changes across pregnancy and the postpartum period (17, 18, 19).


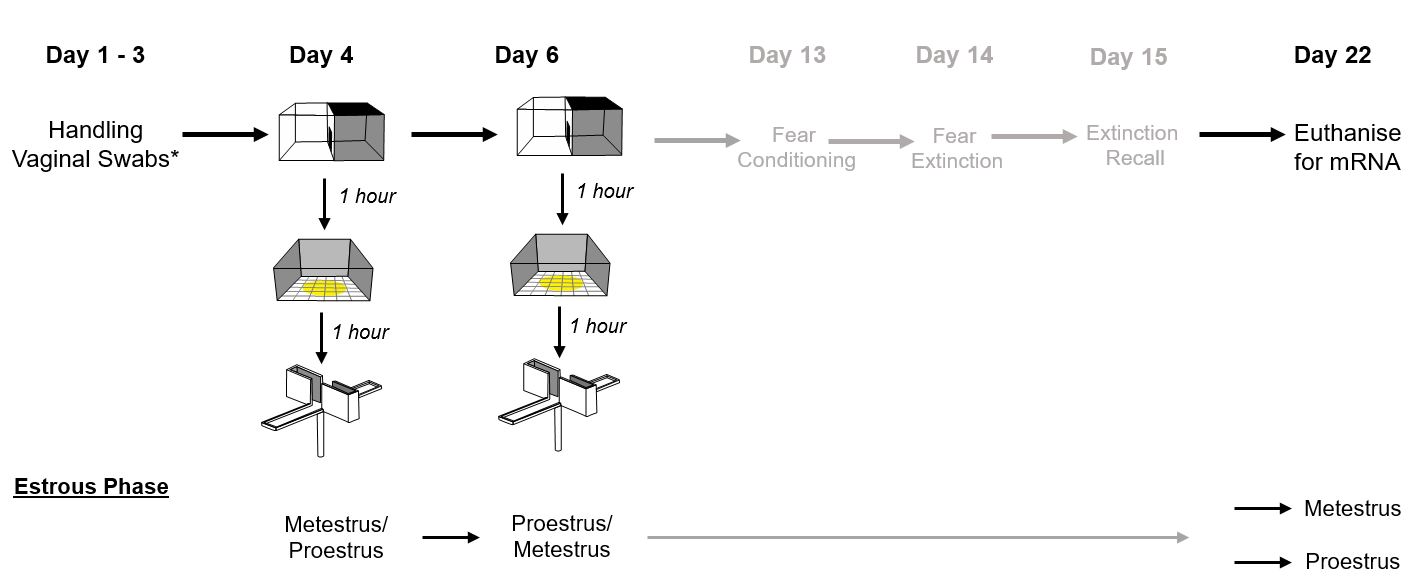


**Figure S1.** Timeline of behavioural testing and estrous phases in Experiment 1. *Vaginal swabs continued daily until the end of the experiment (i.e., Days 1 – 15). Data for fear conditioning, fear extinction, and extinction recall is reported in (13).

***Experiment 2 Procedure***

**Handling.** Rats were handled for 5 min and weighed and swabbed each day for three consecutive days. Rats continued to be swabbed daily until they reached the desired estrous phase for euthanasia.

**Trunk blood collection.** Rats were euthanised following a brief exposure (45s) to carbon dioxide (Co2) and immediately decapitated with a guillotine. Circulating allopregnanolone reaches a maximal increase at 30 min following Co2 inhalation (20) so euthanasia via Co2 was not expected to impact allopregnanolone measurements. Blood was centrifuged within 30 minutes of collection (1000 rcf for 15 min at 4°C) and plasma was stored at -80°C until further analysis. All samples underwent only one freeze-thaw cycle.

**ELISA.** Plasma samples were diluted in a 3-fold dilution (40μL of sample + 80μL of Sample Diluent). This dilution factor was chosen based on a pilot study that tested various dilutions against the standard curve. 50 μL amount of diluted sample was run in duplicate using a commercially available ELISA kit, following manufacturer instructions (OKEH02604, Aviva Systems Biology, USA). Integrated optical density for each sample was measured using an iMARK plate reader (Bio-Rad Laboratories, Hercules, CA) set to a wavelength of 450 nm. This ELISA kit has a sensitivity of 9.5 pg/mL, an intra-assay precision CV of < 3.4%, and an inter-assay prevision CV of < 5.6%.

***Experiment 3 Human Participants***

Participants were recruited across Australia between August 2020 and September 2021 through the university paid research system and community advertisements (online and in-person flyers), and were reimbursed with Flexi Gift cards valued at $5 for every day the questionnaires were completed. There were 152 participants that signed up to the study. Of those who signed up, 29 did not pass the screening assessments due to being pregnant and/or breastfeeding (n = 10), having been pregnant and/or given birth in the past three months (n = 3), being on hormonal contraceptives (n = 5), having medically diagnosed fertility problems or a diagnosed endocrine disorder (n = 5), or having polycystic ovarian syndrome or endometriosis (n = 6). Of the 123 people who were eligible for the study, 66 went on to participate in the study (i.e., sent their next menstrual period date). Of the 66 people who participated in the study, 63 completed the study (i.e., had at least one data point for each timepoint of interest). A power analysis (G Power) indicated that a total sample size of 60 participants would be required for adequate power (1-β = 0.8) to detect small-medium effects (f critical = 0.13; α = 0.05) in a repeated measures design examining within-between interactions, assuming 0.7 correlation among repeated measures.

***Experiment 3 Materials***

**Demographic questionnaire.** Socio-demographic information was assessed via online surveys at the start of the study, including information on menstrual cycle (average menstrual cycle length and onset date of most recent menstrual period) and reproductive history (number of pregnancies, number of biological children, number of non-biological children, and whether they experienced postnatal depression or anxiety, “Have you ever experienced postnatal depression or anxiety before?”). Information on menstrual cycle was used to determine each participant’s expected starting date of the study (i.e., onset of the next menstrual period).

**Patient Health Questionnaire 4 (PHQ-4**). The PHQ-4 is a 4-item scale that measures anxiety and depression symptoms. Participants are asked to rate how often they have experienced each item over the past two weeks using a 4-point scale (0 = *Not at all, 1 = Several Days, 2 = More than half the days, 3 = Nearly every day).* Total scores on the PHQ-4 range from 0 –12. The anxiety subscale is the sum of items 1 and 2 (range from 0 – 6), and the depression subscale is the sum of items 3 and 4 (range from 0 – 6). On each subscale, a score of above 3 indicates clinical anxiety or depression levels.

The wording in the PHQ-4 was adapted to present tense to obtain an ecological momentary assessment. That is, instead of asking participants to rate how often they experienced each item over the past two weeks, participants were asked to rate the extent to which they were experiencing each item in the moment using a 4-point slider scale (e.g., “In this moment, are you feeling nervous, anxious, or on edge?”). The slider scale was also slightly changed to account for the present tense i.e., *Not at all* (0 – 25), *Slightly* (25 – 50), *Moderately* (50 – 75), *Extremely* (75 – 100)*.*

***Experiment 3 Procedure***

Eligible participants were instructed to contact the researcher at the onset of their next menstrual period to partake in the study. A reminder text message was sent a few days before their expected starting date. The day after contacting the researcher (i.e., day after the onset of their menstrual period), participants were sent online questionnaires via scheduled text messages, at three timepoints: 9am, 12pm, and 9pm. Consistent with ecological momentary assessments, the online questionnaires were administered at three timepoints in the day to improve the reliability of the results by minimising the impact of outlier scores (21). Moreover, averaging scores across multiple timepoints would reduce the risk of participant exclusion based on not having at least one data point for each menstrual phase of interest. 9am, 12pm, and 9pm were chosen as representative timepoints of the morning, midday, and evening, respectively. This procedure was repeated 10 and 20 days later (**Figure S2**). Participants were instructed to contact the researcher at the onset of their second menstrual period. The purpose of this was to verify cycle length and to ensure participants received the questionnaires at the correct phases across the second menstrual cycle. After informing the researcher their menstrual period had begun, participants underwent the same testing procedure as the first menstrual cycle (i.e., they were sent three questionnaires a day at three pre-determined phases spaced with 10-day intervals). Participants contacted the researcher at the onset of their third menstrual period to complete the study. The study took approximately 8-weeks (or 2 full menstrual cycles) to complete.

**Determination of menstrual cycle phase****.** The day count method (‘ovulation to ovulation’ backwards counting) was used post-hoc to confirm the menstrual phase (and hence predicted ovarian steroid levels) at the time of each testing phase for each participant (22, 23), which is the recommended method in the absence of measures of ovulation (24). This approach identifies menstrual phases by counting backwards from the date of menses onset (in which 0 = first day of menses) based on the assumption that the luteal phase is relatively fixed at 12-14 days in length due to the predetermined lifespan of the corpus luteum (23). Phases were pre-menstrual (-4 to 0 days), mid-luteal (-9 to -5 days), luteal (-10 to -11 days), ovulation ( -15 to -11 days), and mid-follicular (other days) (**Table S1**). Given that test 1 always occurred the day following menses onset, this test was always during the early-follicular phase (i.e., +1 to +5 days by counting forward from date of menses onset).


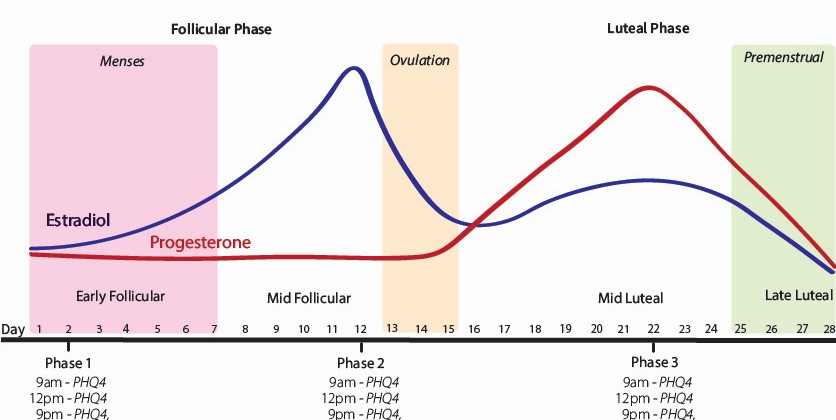


**Figure S2.** Example of the three study phases over a single representative 28-day menstrual cycle. Participants underwent this process over 2 consecutive menstrual phases. Note: PHQ-4 = Patient Health Questionnaire-4.

**Statistical Analysis**

In Experiment 1 (i.e., mRNA expression data only), rats were classified as a statistical outlier and excluded from a target gene analysis if their mRNA expression level was 2 STDEVs away from the group mean. Exclusions included 7 out of 198 data points across all target genes in the BLA (i.e., one nulliparous-proestrus and one primiparous-proestrus rat for α1; one primiparous-proestrus rat for α2; one primiparous-proestrus rat for α5; one nulliparous-metestrus and one primiparous-proestrus rat for ß2; and one nulliparous-metestrus rat for CRH), and 20 out of 234 data points across all target genes in the vHPC (i.e., one nulliparous-metestrus and one nulliparous-proestrus rat for α1; one nulliparous-proestrus and one primiparous-metestrus rat for α2; one nulliparous-metestrus, one nulliparous-proestrus, one primiparous-metestrus, and one primiparous-proestrus rat for α4; two nulliparous-metestrus and one nulliparous-proestrus rats for α5; one nulliparous-metestrus and one nulliparous-proestrus rat for ß2; and three nulliparous-metestrus rats, one nulliparous-proestrus rats, two primiparous-metestrus rats, and one primiparous-proestrus rat for CRH).

In Experiment 2, one nulliparous rat euthanised at proestrus 9am was excluded on the basis that it was a statistical outlier (2.5 STDEVs above the mean) and had a large variability between duplicates (SEM > 100).

**Supplemental Results - Experiments reported in manuscript**

***Experiment 1 OFT***

Additional measures of anxiety-related behaviour were scored on the OFT, including the total time spent freezing, total time spent engaging in unsupported and supported rearing (i.e., standing on hind legs against the walls or without contacting the walls, respectively; 25), and the total number of darts (i.e., rapid movements; 26) There were no group differences on any of these behaviours, and all behaviours remained unchanged from metestrus to proestrus in both nulliparous and primiparous females [data not shown].

***Experiment 1 EPM***

Behaviour on the EPM was analysed with battery of tests entered as a covariate given that half the rats in each reproductive group were tested on the LDB and OFT prior to being tested on the EPM, whereas the other half were tested on the EPM only. Including battery of tests as a covariate in the number of open arm entries led to the main effect of estrous phase to be non-significant *(F*<1)*,* but the estrous phase x reproductive status interaction remained significant (*F*_(1,34)_=4.92, *p*=.03). The main effect of estrous phase was also reduced to non-significance in the anxiety index score *(F*<1)*.* The estrous phase x reproductive status interaction in the number of closed arm entries remained significant (*F*_(1,34)_=6.46, *p*=.02). Battery of tests was not a significant covariate on any measure (*F*s<1).

***Experiment 3 Sample characteristics***

Demographic variables and reproductive history characteristics are presented in **Table 1**. An independent samples *t-*test revealed that groups differed in age (*t*_(61)_=7.22, *p*<.001), such that nulliparous women were younger than parous women. Groups also differed in marital status (χ2(2)=34.55, *p*< 001), educational status (χ2(3)=8.93, *p*=.04), employment status (χ2(2)=16.96, *p*<.001), and race (χ2(3)=21.13, *p*<.001). All parous women had at least one biological child: 42.9% had 1 child, 53.6% had 2 children, and 3.5% had 3 children. 3.5% of parous women had 1 non-biological child and 7.2% of parous women had 2 non-biological children. A large proportion of parous women (64.3%) self-reported having experienced postnatal anxiety and/or depression, which is higher than the 10-20% reported in previous studies (27). As such, it is unclear whether the current sample of parous women are representative of the broader population, and thus, to what extent the findings from the present study will generalise to other samples. However, heightened anxiety and depressive symptoms during the peripartum period are thought to be driven in part by heightened sensitivity to fluctuating ovarian steroids (28). Based on this theory, the current sample of parous women may be particularly hormonally sensitive (and therefore more likely to exhibit menstrual-related changes in anxiety and mood), by virtue of the large proportion who reported postnatal emotional distress. Despite this, no evidence was found for menstrual cycle-related changes in anxiety or mood symptoms in this sample of women. This strongly argues against the possibility that the null findings were driven by sample specific factors. Nonetheless, future studies should specifically examine the impact of menstrual cycle on anxiety and mood symptoms in a broader sample of women, including those with and without a history of peripartum anxiety and/or depression.

***Experiment 3 Menstrual cycle characteristics***

Menstrual cycle characteristics are presented in **Table S1.** An independent samples *t-*test revealed that groups differed in their menstrual cycle length (*t*_(107)_=2.86, *p*<.01), such that nulliparous women had a longer menstrual cycle than parous women. This effect remained significant even after removing outliers from the nulliparous group (i.e., 3 cycle lengths < 36 days; *t*_(104)_=2.35, *p=*.02). Importantly, the number of menstrual phases identified using the ‘ovulation to ovulation’ method did not differ in phase 2 (χ2(4)=2.70, *p*=.64) or phase 3 (χ2(6)=8.72, *p*=.18) between nulliparous and parous women.

***Experiment 3 PHQ-4 Anxiety***

Anxiety scores were analysed with age and menstrual cycle length entered as covariates given that there were group differences on these variables. Age and cycle length were not significant covariates (*F*s<1) and their inclusion did not change the pattern of means; although, the main effect of menstrual cycle (*F*< 1) and the menstrual phase x reproductive status interaction were reduced to non-significance (*F*_(2,118)_=2.27, *p=*.11). In parous women, the amount of parity (i.e., whether parous women had one or more than one biological child) was entered as a covariate in an exploratory analysis. The amount of parity was not a significant covariate (*F*_(1,26)_=2.91, *p*=.10), and did not impact the pattern of results (non-significant main effect of menstrual cycle and menstrual cycle x parity interaction; *F’s*<1). In addition, the time since the birth of their youngest child was entered as a covariate in an exploratory analysis. The time since birth was not a significant covariate (*F*<1), and did not impact the pattern of results (non-significant main effect of menstrual cycle and menstrual cycle x parity interaction; *F’s*<1).

***Experiment 3 PHQ-4 Depression***

Depression scores were analysed with age and menstrual cycle length entered as covariates given that there were group differences on these variables. Age and cycle length were not significant covariates (largest *F*=1.08) and their inclusion did not change the pattern of means; although, the main effect of menstrual cycle was no longer significant (*F*_(2,118)_=.33, *p*=.68), but the menstrual phase x reproductive status interaction remained significant (*F*_(2,118)_=4.09, *p*=.03), illustrating that the impact of menstrual phase on mood was moderated by reproductive status even after accounting for age and cycle length differences.

In parous women, the amount of parity did not impact the pattern of results (non-significant main effect of menstrual cycle and menstrual cycle x parity interaction; *Fs*<1), however amount of parity was a significant covariate such that primiparous women self-reported higher depression scores than multiparous women (*F*_(1,26)_=5.78, *p*=.02). In addition, the time since birth was not a significant covariate (*F*_(1,23)_=1.39, *p*=.25), and did not impact the pattern of results (non-significant main effect of menstrual cycle and menstrual cycle x parity interaction; *F*_(2,46)_=1.00, *p*=.37).

|  | **Nulliparous women** | | **Parous women** | | | | | **Statistic** |  |
| --- | --- | --- | --- | --- | --- | --- | --- | --- | --- |
|  | **M (SD)** | **Range** | **M (SD)** | | **Range** | | |  |  |
| **Menstrual Cycle Length (days)** | 30.54 (3.61) | 21 - 42 | | 28.71 (2.91) | | 23 - 36 | *t*(107) = 2.86 , *p* = .005 | | |
|  | **n** | **%** | **n** | | **%** | | |  |  |
| **Phase 2** |  |  |  | |  | | | χ2(4) = 2.70, *p* = .64 |  |
| Luteal  Ovulation | 1  6 | 1.4  8.6 | 0  9 | | 0  16.1 | | |  |  |
| Mid-Follicular | 53 | 75.7 | 38 | | 67.9 | | |  |  |
| No data point | 5 | 7.1 | 4 | | 7.1 | | |  |  |
| No menses onset date | 5 | 7.1 | 5 | | 8.9 | | |  |  |
| **Phase 3** |  |  |  | |  | | | χ2(6) = 8.72, *p* = .18 |  |
| Pre-Menstrual | 4 | 5.7 | 8 | | 14.3 | | |  |  |
| Mid-Luteal | 24 | 34.3 | 26 | | 46.4 | | |  |  |
| Luteal | 20 | 28.6 | 9 | | 16.1 | | |  |  |
| Ovulation  Mid-Follicular | 9  3 | 12.9  4.3 | 4  0 | | 7.1  0 | | |  |  |
| No data point | 5 | 7.1 | 4 | | 7.1 | | |  |  |
| No menses onset date | 5 | 7.1 | 5 | | 8.9 | | |  |  |

**Table S1.** Menstrual cycle length characteristics and predicted menstrual phases during testing of sample.

*Note:* Phases were estimated using the ‘ovulation to ovulation’ method (22, 23). This approach identifies menstrual phases by counting backwards from the date of menses onset (in which 0 = first day of menses). Phases were: pre-menstrual (-4 to 0 days), mid-luteal (- 9 to -5 days), luteal (-10 to -11 days), ovulation ( -15 to -11 days), and mid-follicular (other days). Given that Test 1 always occurred the day following menses onset, this test was always during the menstrual phase (i.e., +1 to +5 days by counting forward from date of menses onset)

**Supplemental Experiment - Methods, Materials, and Results**

In Supplementary Experiment 1, we aimed to disentangle the role of pregnancy versus the maternal experience (i.e., exposure to pups) on the dissociable impact of estrous cycle on anxiety-like behaviour following reproductive experience. As per (29), primiparous rats either experienced both pregnancy and pup exposure (i.e., as in Experiment 1) or had their pups permanently removed within 24h of parturition (i.e., experienced pregnancy only). Replicating Experiment 1, it was expected that primiparous rats that experienced pregnancy and pup exposure would show comparable anxiety-like behaviour irrespective of the estrous phase in which they were tested. If pregnancy rather than maternal experience (i.e., lactation and pup exposure) causes estrous-independent anxiety-like behaviour, then primiparous rats that experienced pregnancy, but not pup exposure, should also show comparable anxiety-like behaviour irrespective of the estrous phase in which they were tested.

***Animal Subjects***

Experimentally naïve primiparous Sprague Dawley rats obtained from the ARC were used in this experiment. Mating in primiparous rats occurred at the ARC as previously described, with the only exception being that primiparous rats either remained with their pups until weaning (primiparous, n = 16) or had their pups permanently removed within 24 hours of parturition (primiparous-pregnancy-only, n = 16). Upon arrival at UNSW, rats were housed under the same conditions as described. Primiparous and primiparous-pregnancy-only rats were age-matched for each experiment and underwent behavioural testing at approximately 6 months of age (24 weeks). Behavioural testing began approximately 1 month postweaning in primiparous rats (or 2 months after parturition in primiparous-pregnancy-only rats).

***Procedure***

**Handling.** Rats were handled for 5 min and weighed and swabbed each day for three consecutive days prior to any experimental procedures.

**Battery of tests.** Rats were tested twice on a battery of tests including the LDB and EPM over two testing days, once during metestrus and once during proestrus. Given that the estrous phase is typically 4 days long, there was a 2-day rest period between testing days. The battery of tests procedure was the same as Experiment 1. However, unlike Experiment 1, the OFT was not included in this experiment given that estrous cycle did not influence anxiety-like behaviour in nulliparous rats on this task.

***LDB.*** The LDB procedures were the same as those described in Experiment 1, and occurred during 1200h-1300h.

***EPM.*** One hour after the LDB, rats were tested on the EPM. The EPM procedures were the same as those described in Experiment 1, conducted under high light intensity (300 lux).

***Statistical analysis***

Separate mixed model ANOVAs with the between-subjects factor of pup exposure (primiparous or primiparous-pregnancy only) and the within-subjects factor of estrous phase (metestrus and proestrus) were used to assess group differences in anxiety-like behaviour on the LDB and EPM. Planned paired samples *t*-tests with Bonferroni correction (*p* = .025) were used to assess estrous cycle effects on measures of anxiety-like behaviour in primiparous and primiparous-pregnancy-only groups separately.

## ***Results***

**LDB.** There were no main effects of estrous phase or pup exposure, and no estrous phase x pup exposure interaction in the latency to enter the light compartment, time spent in the light compartment, or number of entries in the light compartment (see **Figure S2,** largest *F*_(1,30)_=2.12, *p*=.16). Replicating Experiment 1, primiparous rats showed comparable behaviour on all measures from metestrus to proestrus (*ts*<1). Likewise, all behaviours remained unchanged from metestrus to proestrus in primiparous-pregnancy-only rats (largest *t*_(15)_=1.80, *p*=.09).

**EPM.** There were no main effects of estrous phase or pup exposure, and no interaction between factors in the time spent in the open arms or number of open arm entries (see **Figure** **S2**; largest *F_(_*_1,30)_=2.94, *p*=.10). There was no main effect of estrous phase or estrous phase x pup exposure interaction in the number of closed arm entries, however there was a significant main effect of pup exposure whereby primiparous rats made more closed arm entries than primiparous-pregnancy-only rats (*F*_(1,30)_=4.55, *p*=.04). Primiparous rats showed a decrease in the number of closed arm entries from metestrus to proestrus (*t*_(15)_=2.41, *p*=.03), however replicating Experiment 1, all other measures remained unchanged (largest *t*_(15)_=1.15, *p*=.27). In primiparous-pregnancy-only rats, all behaviours remained unchanged from metestrus to proestrus (largest *t*_(15)_=1.32, *p*=.21).

There were no main effects of estrous phase or pup exposure, and no interaction between factors on the anxiety index ratio (largest *F_(_*_1,30)_=1.21, *p*=.28). Replicating Experiment 1, the anxiety index remained unchanged from metestrus to proestrus in primiparous rats (*t*s<1). Likewise, no changes were observed in primiparous-pregnancy-only rats (*t*s<1).

*
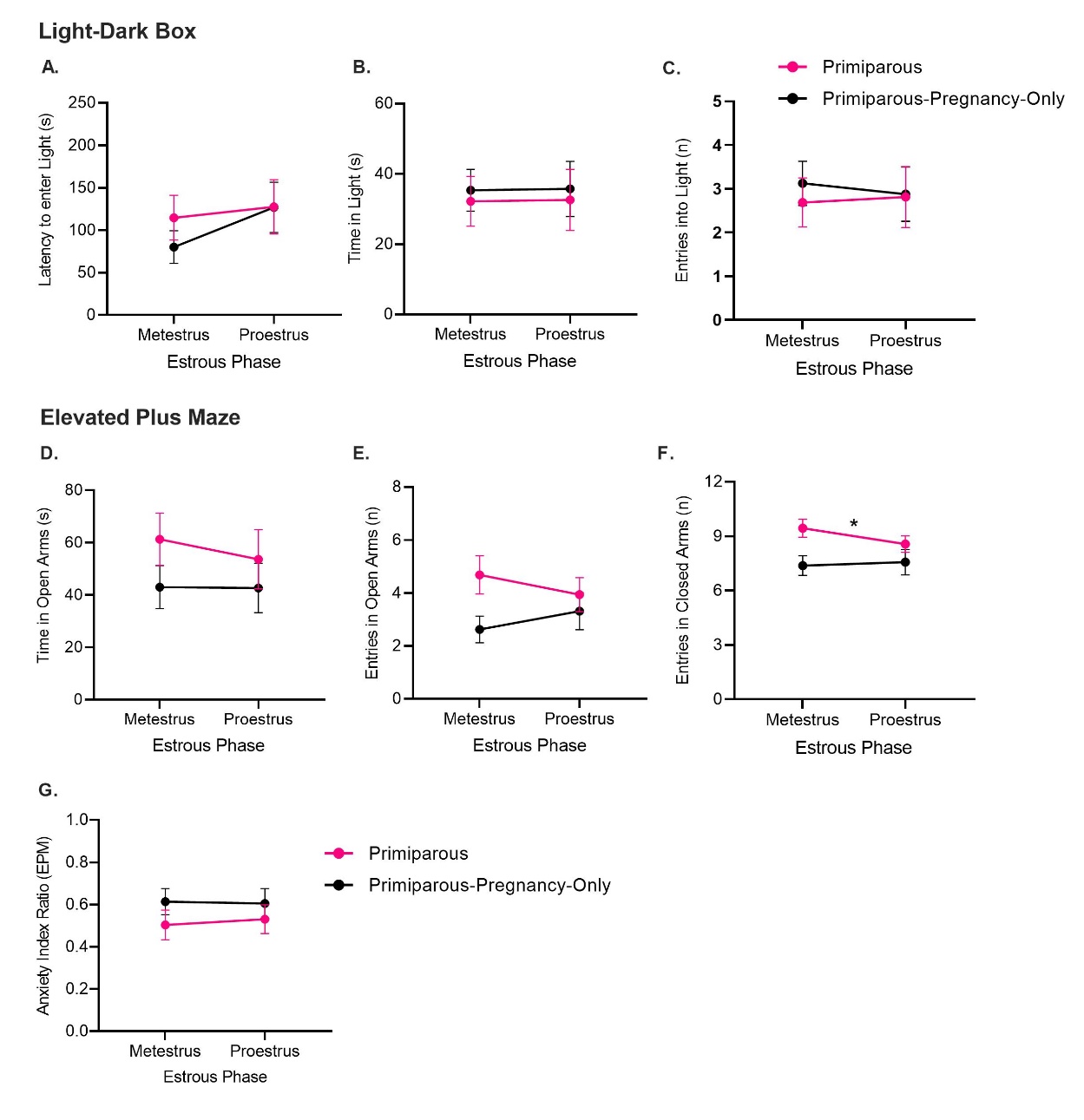
*

**Figure S3.** Primiparous (n = 16) and primiparous-pregnancy-only (n = 16) rats were tested twice on the light-dark box (LDB) and elevated plus maze (EPM); once during metestrus, and once during proestrus, with the estrous phase at test being counterbalanced. (**A)** Mean (±SEM) latency to enter the light compartment in the LDB. (**B)** Mean (±SEM) time spent in the light compartment. **(C)** Mean (±SEM) number of entries into the light compartment. **(D)** Mean (±SEM) time spent in open arms of the EPM. (**E)** Mean (±SEM) number of entries in open arms. **(F)** Mean (±SEM) number of entries in the closed arms. *Primiparous rats > Primiparous-Pregnancy-only rats (*p* < .05). **(G)** Mean (±SEM) anxiety index ratio.

**Supplemental References**

1. Molina-Hernandez M, Tellez-Alcantara NP, Olivera-Lopez JI, Jaramillo MT. Estrous cycle variation in anxiolytic-like effects of topiramate in Wistar rats in two animal models of anxiety-like behavior. Pharmacology Biochemistry and Behavior. 2013;103(3):631-6.

2. Pentkowski NS, Litvin Y, Blanchard DC, Blanchard RJ. Effects of estrus cycle stage on defensive behavior in female Long-Evans hooded rats. Physiol Behav. 2018;194:41-7.

3. Rey CD, Lipps J, Shansky RM. Dopamine D1 receptor activation rescues extinction impairments in low-estrogen female rats and induces cortical layer-specific activation changes in prefrontal-amygdala circuits. Neuropsychopharmacology. 2014;39(5):1282-9.

4. Sayin A, Derinoz O, Yuksel N, Sahin S, Bolay H. The effects of the estrus cycle and citalopram on anxiety-like behaviors and c-fos expression in rats. Pharmacol Biochem Behav. 2014;124:180-7.

5. Mora S, Dussaubat N, Diaz-Veliz G. Effects of the estrous cycle and ovarian hormones on behavioral indices of anxiety in female rats. Psychoneuroendocrinology. 1996;21(7):609-20.

6. Becker JB, Arnold AP, Berkley KJ, Blaustein JD, Eckel LA, Hampson E, et al. Strategies and methods for research on sex differences in brain and behavior. Endocrinology. 2005;146(4):1650-73.

7. Frye CA, Petralia SM, Rhodes ME. Estrous cycle and sex differences in performance on anxiety tasks coincide with increases in hippocampal progesterone and 3alpha,5alpha-THP. Pharmacol Biochem Behav. 2000;67(3):587-96.

8. Guillén-Ruiz G, Cueto-Escobedo J, Hernández-López F, Rivera-Aburto LE, Herrera-Huerta EV, Rodríguez-Landa JF. Estrous cycle modulates the anxiogenic effects of caffeine in the elevated plus maze and light/dark box in female rats. Behav Brain Res. 2021;413:113469.

9. Kastenberger I, Schwarzer C. GPER1 (GPR30) knockout mice display reduced anxiety and altered stress response in a sex and paradigm dependent manner. Horm Behav. 2014;66(4):628-36.

10. Koonce CJ, Frye CA. Progesterone facilitates exploration, affective and social behaviors among wildtype, but not 5α-reductase Type 1 mutant, mice. Behav Brain Res. 2013;253:232-9.

11. Scholl JL, Afzal A, Fox LC, Watt MJ, Forster GL. Sex differences in anxiety-like behaviors in rats. Physiol Behav. 2019;211:112670.

12. Walf AA, Koonce C, Manley K, Frye CA. Proestrous compared to diestrous wildtype, but not estrogen receptor beta knockout, mice have better performance in the spontaneous alternation and object recognition tasks and reduced anxiety-like behavior in the elevated plus and mirror maze. Behav Brain Res. 2009;196(2):254-60.

13. Pestana JE, McCutcheon TB, Harmon-Jones SK, Richardson R, Graham BM. Maternal Experience Does Not Predict Fear Extinction and Anxiety-Like Behaviour in Primiparous Rats Post-weaning. Front Glob Womens Health. 2021;2:742337.

14. Byrnes EM, Lee JO, Bridges RS. Alterations in GABA(A) receptor alpha2 subunit mRNA expression following reproductive experience in rats. Neuroendocrinology. 2007;85(3):148-56.

15. Bakshi VP, Kalin NH. Corticotropin-releasing hormone and animal models of anxiety: gene-environment interactions. Biol Psychiatry. 2000;48(12):1175-98.

16. Young CE, Tong Q. Corticotropin Releasing Hormone Signaling in the Bed Nuclei of the Stria Terminalis as a Link to Maladaptive Behaviors. Front Neurosci. 2021;15:642379.

17. Bloch M, Daly RC, Rubinow DR. Endocrine factors in the etiology of postpartum depression. Compr Psychiatry. 2003;44(3):234-46.

18. Klampfl SM, Bosch OJ. Mom doesn't care: When increased brain CRF system activity leads to maternal neglect in rodents. Front Neuroendocrinol. 2019;53:100735.

19. Voltolini C, Petraglia F. Neuroendocrinology of pregnancy and parturition. Handb Clin Neurol. 2014;124:17-36.

20. Barbaccia ML, Roscetti G, Trabucchi M, Mostallino MC, Concas A, Purdy RH, et al. Time-dependent changes in rat brain neuroactive steroid concentrations and GABAA receptor function after acute stress. Neuroendocrinology. 1996;63(2):166-72.

21. Shiffman S, Stone AA, Hufford MR. Ecological momentary assessment. Annu Rev Clin Psychol. 2008;4:1-32.

22. Edler C, Lipson SF, Keel PK. Ovarian hormones and binge eating in bulimia nervosa. Psychol Med. 2007;37(1):131-41.

23. Eisenlohr-Moul TA, Kaiser G, Weise C, Schmalenberger KM, Kiesner J, Ditzen B, et al. Are there temporal subtypes of premenstrual dysphoric disorder?: using group-based trajectory modeling to identify individual differences in symptom change. Psychol Med. 2020;50(6):964-72.

24. Schmalenberger KM, Tauseef HA, Barone JC, Owens SA, Lieberman L, Jarczok MN, et al. How to study the menstrual cycle: Practical tools and recommendations. Psychoneuroendocrinology. 2021;123:104895.

25. Sturman O, Germain PL, Bohacek J. Exploratory rearing: a context- and stress-sensitive behavior recorded in the open-field test. Stress. 2018;21(5):443-52.

26. Gruene TM, Roberts E, Thomas V, Ronzio A, Shansky RM. Sex-specific neuroanatomical correlates of fear expression in prefrontal-amygdala circuits. Biological Psychiatry. 2015;78(3):186-93.

27. Pawluski JL, Lonstein JS, Fleming AS. The Neurobiology of Postpartum Anxiety and Depression. Trends Neurosci. 2017;40(2):106-20.

28. Bloch M, Schmidt PJ, Danaceau M, Murphy J, Nieman L, Rubinow DR. Effects of gonadal steroids in women with a history of postpartum depression. Am J Psychiatry. 2000;157(6):924-30.

29. Pawluski JL, Lieblich SE, Galea LA. Offspring-exposure reduces depressive-like behaviour in the parturient female rat. Behav Brain Res. 2009;197(1):55-61.
